# Supplementary material for: Identification and expression analysis of the GDSL esterase/lipase family genes, and the characterization of SaGLIP8 in Sedum alfredii Hance under cadmium stress
Source: PeerJ. 2019 Apr 16;7:e6741. doi: 10.7717/peerj.6741 (PMC6474334; doi:10.7717/peerj.6741)
Supplement: Table S6 — The pictures of motif were downloaded from online website MEME ( http://meme-suite.org/tools/meme). Sa, Sedum alfredii. [file peerj-07-6741-s006.docx]

**Table S6 Conserved motifs identified in the SaGLIP proteins**

| **Motif No.** | **Motif consensus sequence** | **E value** | **No. of *SaGLIP* proteins** | **Length (amino acids)** | **Relative Entropy** | **Bayes Threshold** |
| --- | --- | --- | --- | --- | --- | --- |
| 1 | 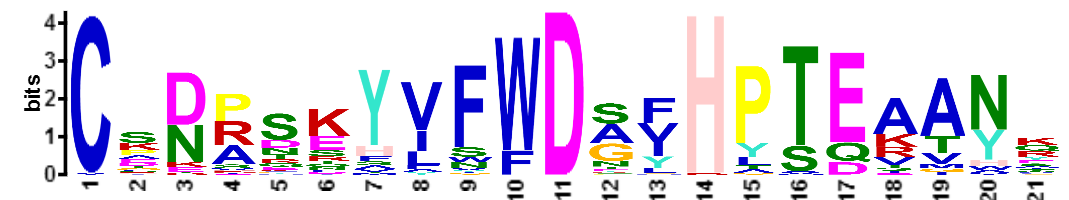 | 8.6e-977 | 80 | 21 | 58.6 | 8.47137 |
| 2 | 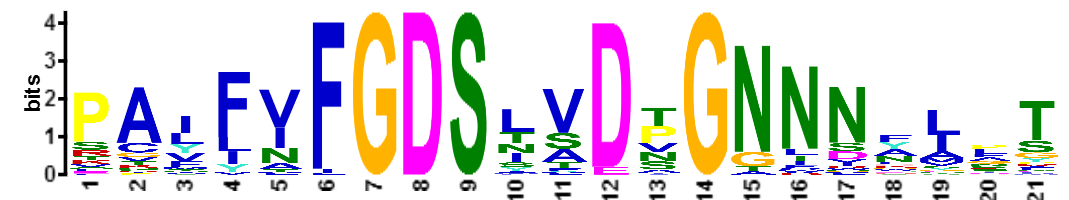 | 7.7e-758 | 80 | 21 | 49.9 | 8.72356 |
| 3 | 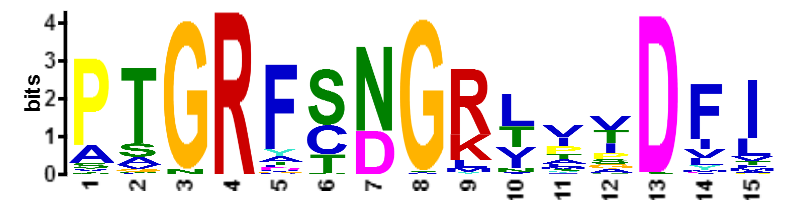 | 9.4e-608 | 79 | 15 | 41 | 8.75204 |
| 4 | 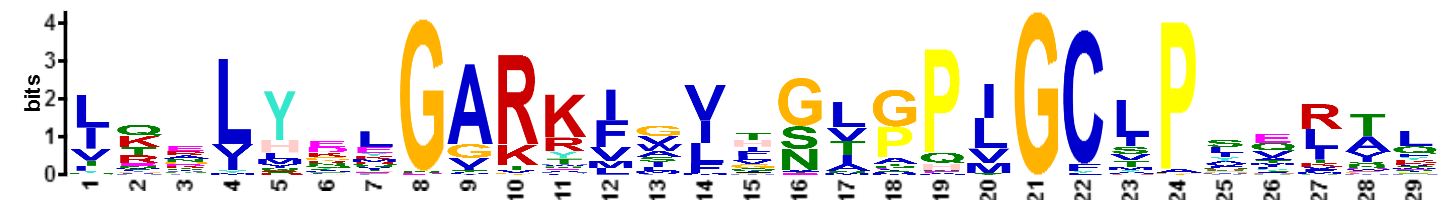 | 3.9e-880 | 80 | 29 | 59.1 | 8.43848 |
| 5 | 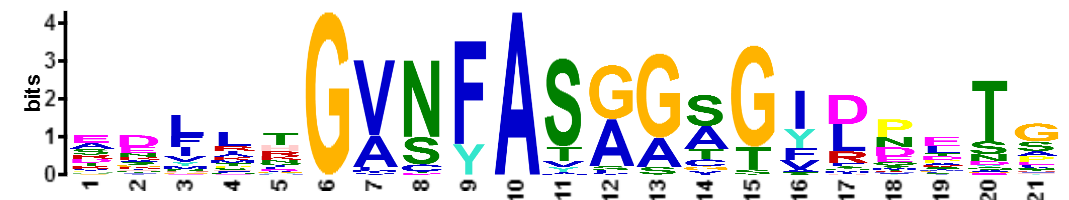 | 8.2e-574 | 79 | 21 | 42.8 | 8.47499 |
| 6 | 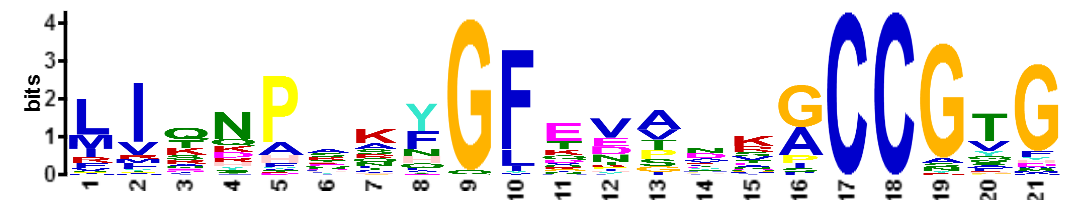 | 8.5e-535 | 70 | 21 | 44.9 | 8.508 |
| 7 | 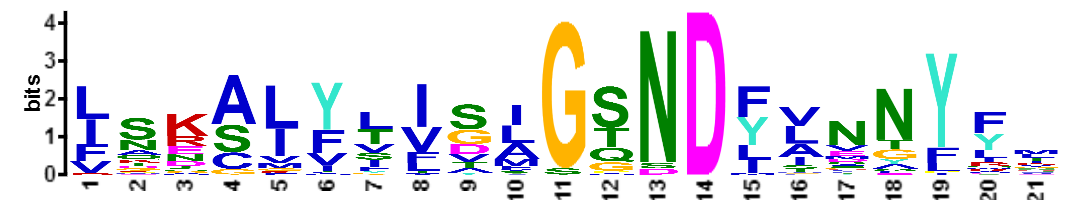 | 1.5e-493 | 72 | 21 | 42.7 | 10.0304 |
| 8 | 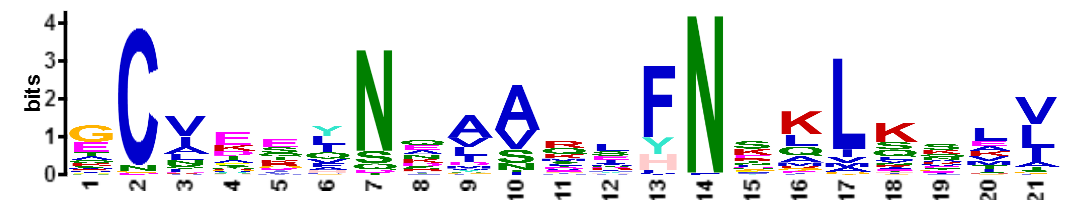 | 9.3e-401 | 79 | 21 | 35.5 | 8.72787 |
| 9 | 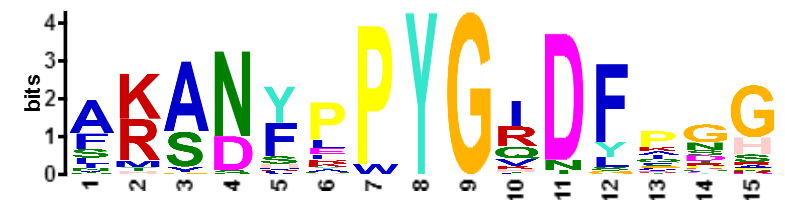 | 3.0e-362 | 58 | 15 | 38.9 | 9.60239 |
| 10 | 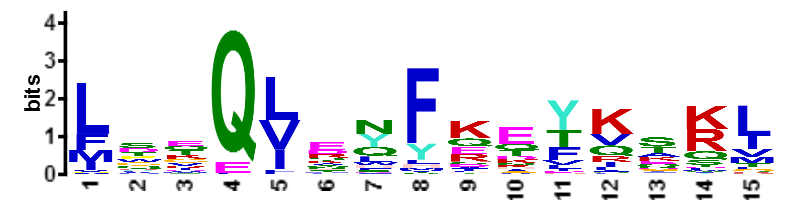 | 1.50E-245 | 79 | 15 | 26.3 | 8.49918 |
| 11 | 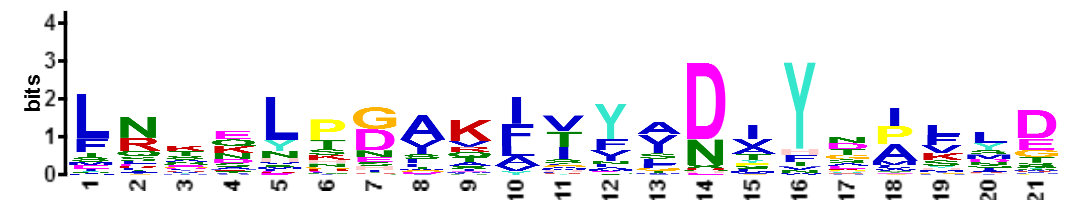 | 7.70E-239 | 70 | 21 | 31.4 | 8.508 |
| 12 | 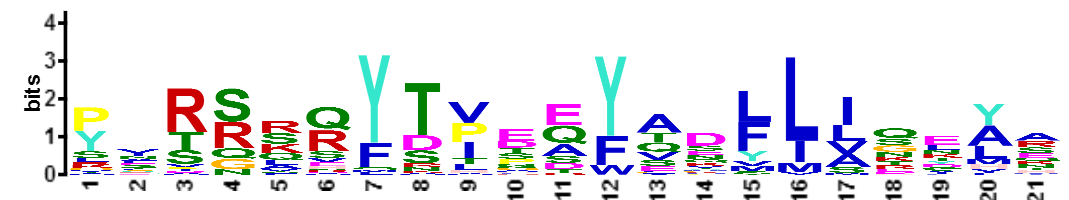 | 1.30E-192 | 49 | 21 | 36.9 | 8.86353 |
| 13 | 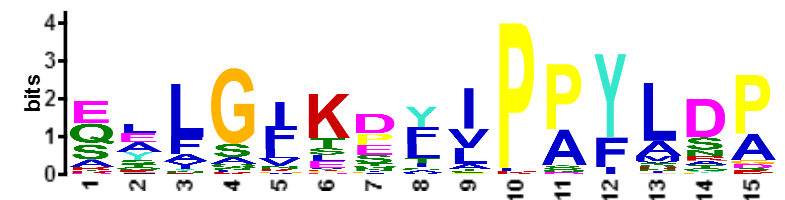 | 2.20E-175 | 49 | 15 | 31.8 | 8.8877 |
| 14 | 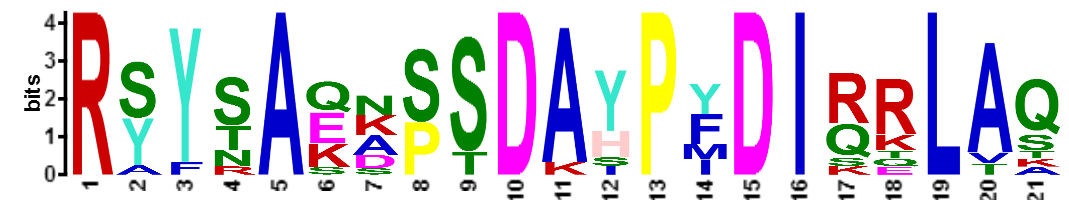 | 1.50E-96 | 12 | 21 | 68.8 | 12.3116 |
| 15 | 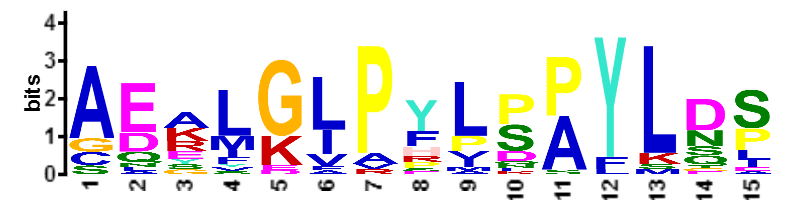 | 5.10E-63 | 24 | 15 | 35.5 | 11.3355 |
| 16 | 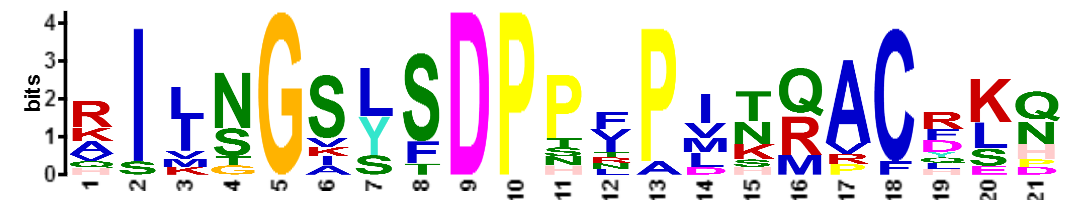 | 2.50E-47 | 11 | 21 | 59.1 | 11.6926 |
| 17 | 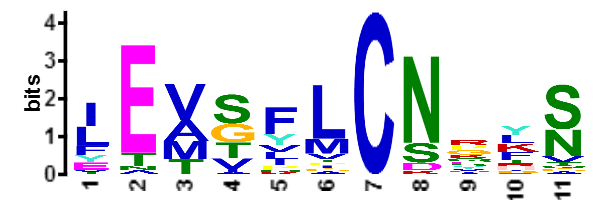 | 1.10E-42 | 30 | 11 | 25.2 | 9.85392 |
| 18 | 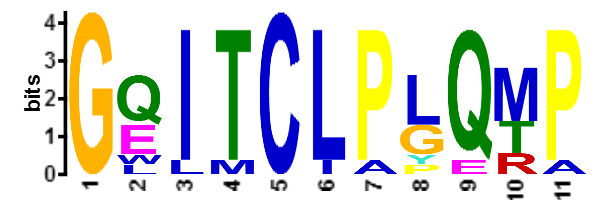 | 7.90E-34 | 10 | 11 | 39.9 | 12.4586 |
| 19 | 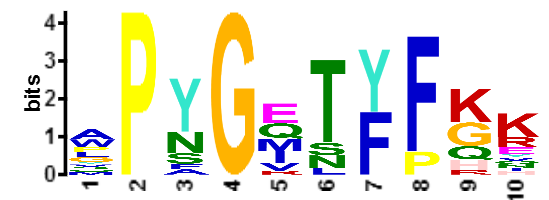 | 4.90E-23 | 18 | 10 | 27.7 | 11.5188 |
| 20 | 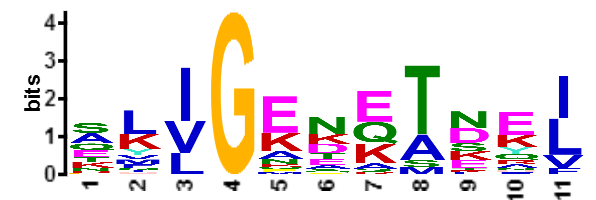 | 3.00E-21 | 30 | 11 | 23.2 | 11.2045 |
| 21 | 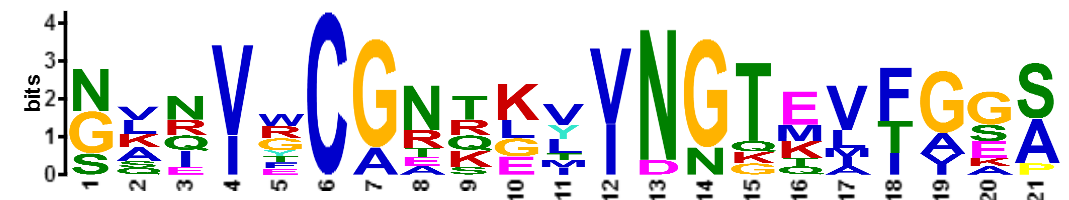 | 1.30E-18 | 10 | 21 | 53.9 | 11.7265 |
| 22 | 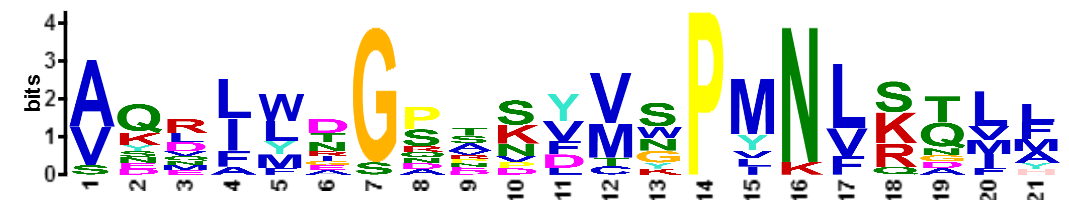 | 6.60E-18 | 12 | 21 | 48.9 | 10.8706 |
| 23 | 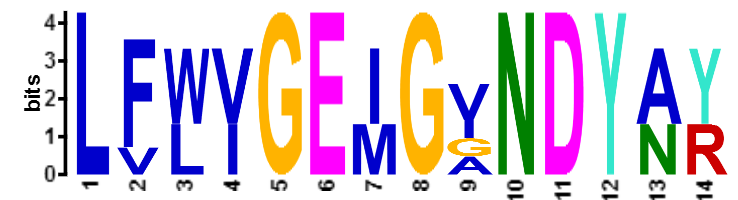 | 1.20E-11 | 5 | 14 | 51.5 | 12.7549 |
