# Supplementary figures and images for: Identification and expression analysis of the GDSL esterase/lipase family genes, and the characterization of SaGLIP8 in Sedum alfredii Hance under cadmium stress
Source: PeerJ. 2019 Apr 16;7:e6741. doi: 10.7717/peerj.6741 (PMC6474334; doi:10.7717/peerj.6741)

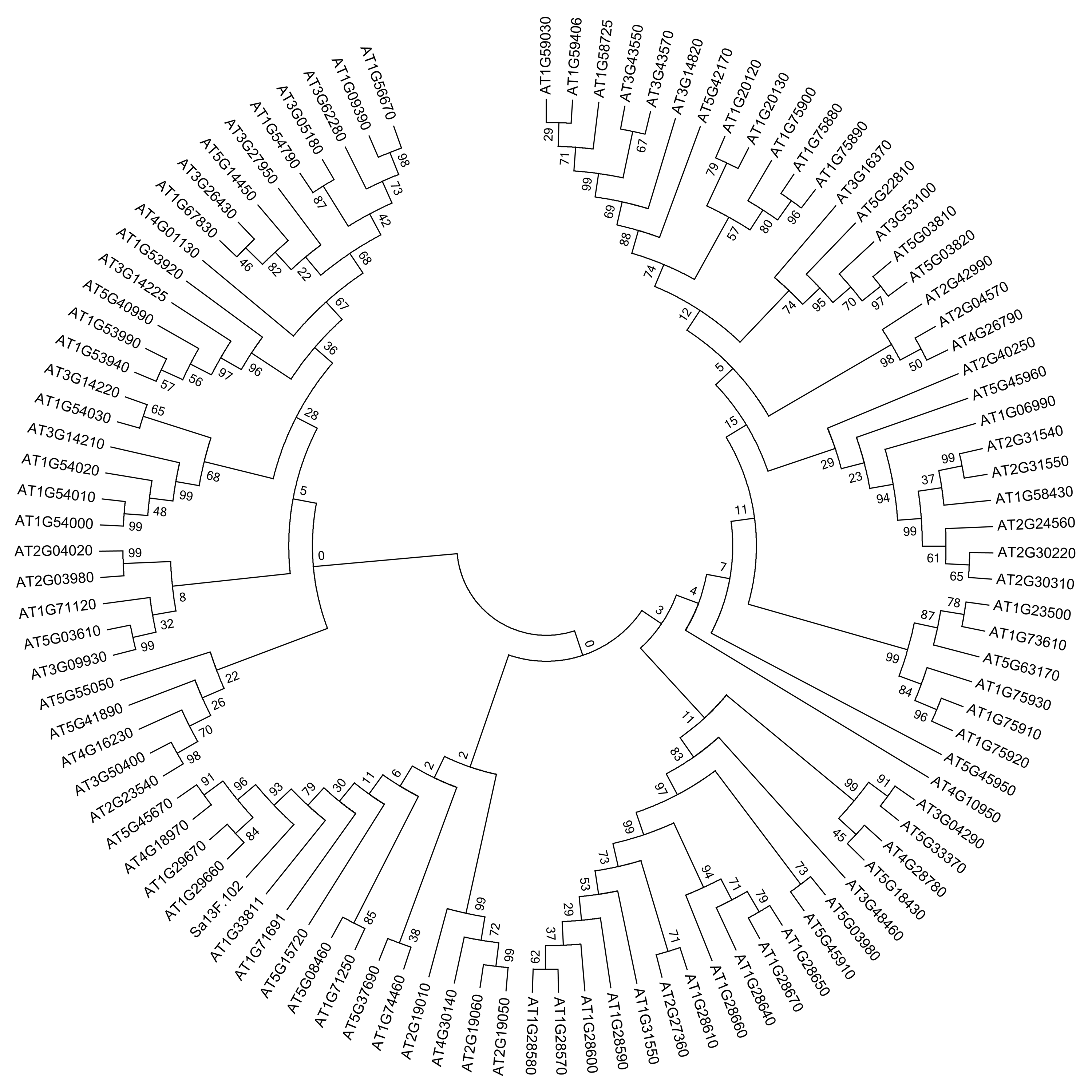

Supplement: Figure S1 — The tree was generated with ClustalW and MEGA 5.0 software using the Neighbor-joining method. The number of bootstrap replications is 1,000. [file peerj-07-6741-s009.png]

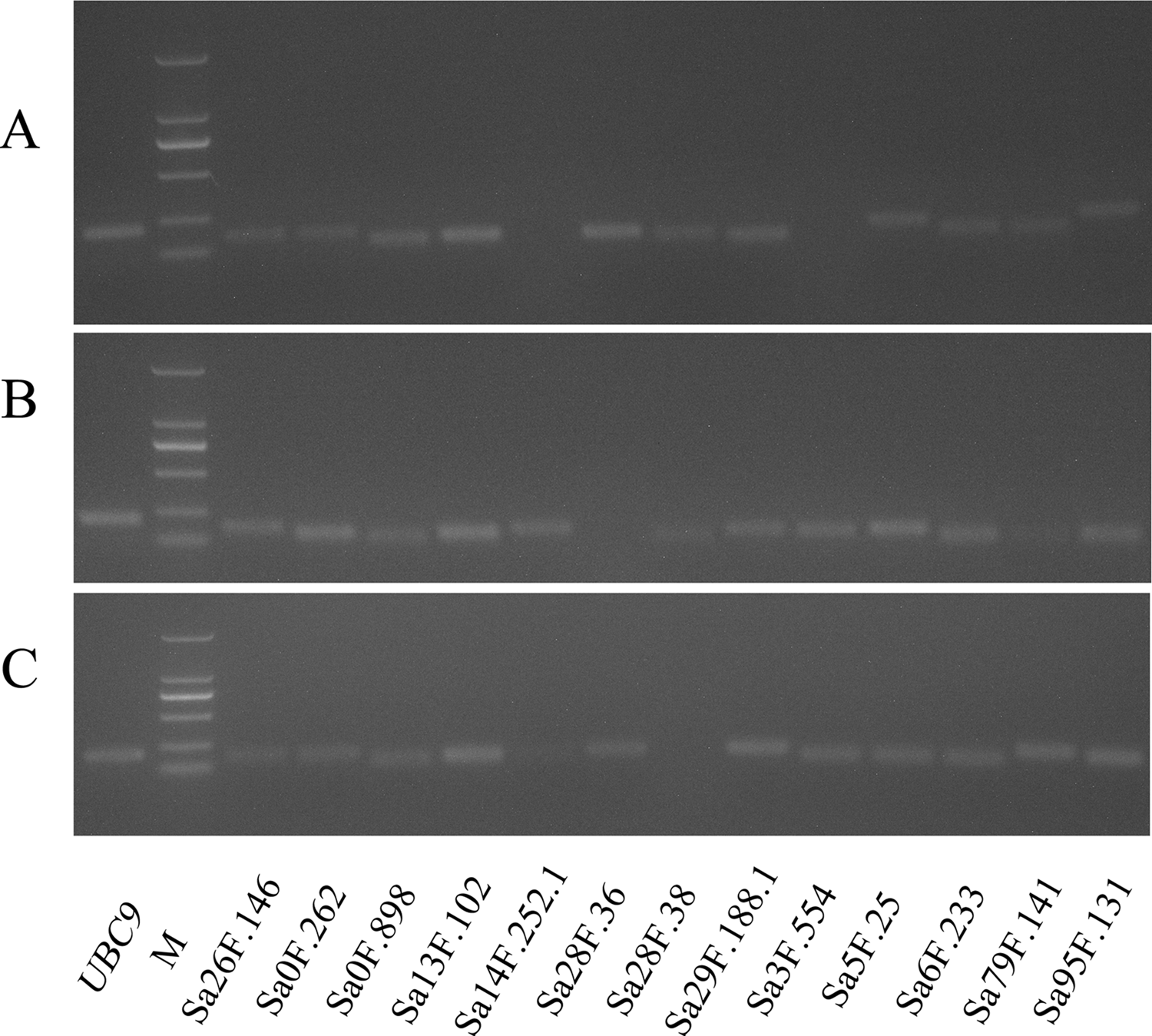

Supplement: Supplemental Information 10 — (A) root; (B) stem; (C) leaf. UBC9 is reference gene of S. alfredii. M represents 2,000 bp DNA ladder marker. Relative expression values were calculated by Z-score normalization. Green and red showed the low and high expression levels, respectively. The names of the samples are exhibited at the bottom. [file peerj-07-6741-s010.png]
